# Ameloblastoma and Intracranial Involvement: The Current Challenge of the Radical Surgical Treatment. Comprehensive Review of the Literature and Institution experience

**DOI:** 10.1007/s12663-021-01643-9

**Published:** 2021-09-24

**Authors:** Daniele Armocida, Luigi Valentino Berra, Resi Pucci, Andrea Battisti, Marco Della Monaca, Valentino Valentini, Antonio Santoro

**Affiliations:** 1 Human Neurosciences Department Neurosurgery Division, Sapienza University of Rome, Rome, Italy; 2 Department of Oral and Maxillofacial Sciences, Sapienza University of Rome, Via Caserta 6, 00161 Roma, Italy; 3 Oncological and Reconstructive Maxillo-Facial Surgery Unit. Policlinico, Umberto I Di Roma, Roma, Italy

**Keywords:** Ameloblastoma, Intracranial ameloblastoma, Combined approach, Recurrence, Craniofacial tumor

## Abstract

**Background:**

Ameloblastoma (AMBL) is an odontogenic tumor, considered to be benign, but aggressive, whose principal risk is a recurrence. The growth can be enormous, and it can extend into the intracranial compartment with serious consequences.

**Purpose:**

The intracranial involvement of AMBL is rare, and it may require an extensive surgery. Although it is a rare condition for the neurosurgeon to treat, knowing this condition can lead to a significant increase in survival for these patients.

**Methods:**

A case of a 56-year-old woman presented with a history of recurrent left maxilla AMBL with intracranial extension and dural involvement of the anterior and medial cranial fossa is reported, followed by a systematic review of the literature with the aim to identify the best surgical treatment.

**Results:**

A total of 32 cases were included in the qualitative analysis. Management is varied and often not described, resulting in an almost complete lack of information and indications for treatment. Radical surgery tends to yield the best outcomes, and it is recommended to have adequate surgical margins when possible.

**Conclusions:**

Intracranial involvement from AMBL compartment is an uncommon manifestation of this rare pathology, but which deserves to be treated in a multidisciplinary way in order to ensure maximum surgical radicality. Recurrence reflects failure of the primary surgical resection. If recurrence is the major consideration, surgeons are encouraged to select radical surgery. Whenever a follicular-type maxillary AMBL is diagnosed, it is advisable to check for intracranial spreading and distant metastases during follow-up.

## Introduction

Ameloblastoma (AMBL) is an odontogenic tumor, considered to be a benign, but aggressive, whose principal risk is a recurrence [1, 2]. The growth can be enormous, and it can extend into the intracranial compartment with serious consequences. Although classified as a benign tumor, AMBL is also the most common odontogenic tumor of epithelial origin with severe clinical implications [3]. AMBL has a locally aggressive growth pattern, spreading to the base of the skull, paranasal sinuses, infratemporal fossa, pterygopalatine fossa, parapharyngeal space, and orbit [4]. The principal risk is the local recurrence, especially the case of closed margins is less than 0.5 mm. (1 cm) [5]. About 2% of cases evolve in a malignant form [6]. Brain involvement can lead to life-threatening complications as, intracranial hypertension, severe neurological deficits up to the patient's death. Although it is a rare condition for the maxillofacial surgeons and neurosurgeons to treat, the knowledge of this condition can significantly increase survival for these patients. The management of these patients is complex, there are no guidelines or international recommendations and often in many cases it is possible to perform only a palliative surgery without being able to guarantee the surgical radicality that, however, allows to increase the survival of these patients. For these reasons we decided to implement our experience with a comprehensive review of the literature on the treatment of this rare pathology when it involves the intracranial compartment.

We perform an accurate analysis of all cases reported in literature and present a case of a 56-year-old woman presented with a history of recurrent left maxilla AMBL with intracranial extension and dural involvement of the anterior and medial cranial fossa in order to retrieve information regarding clinical features and surgical treatment.

## Materials and Methods

### Eligibility Criteria

A literature review was performed, and the following inclusion criteria were adopted: 1) studies that address the topic of AMBL that involves intracranial compartment; 2) review or collection of cases, case series and case report that describe diagnosis, treatment, surgical approach and prognosis of patients.

Exclusion criteria were: (1) short reports or letter; (2) papers that study AMBL without a specific treatment of intracranial portion and (3) articles not written in English.

### Information Sources and Search

The study was conducted in accordance with PRISMA statements. The English literature was systematically investigated using MEDLINE, the NIH Library, PubMed, and Google Scholar. The last search date was August 20, 2019.

Search terms included: "Ameloblastoma" in combination with "brain" or "intracranial." All cases of AMBL with intracranial involvement were included. Backward citation tracking was applied to identify articles not retrieved by electronic searches. Two independent authors (D.A. and L.V.B.) conducted the first research of electronic media and any discordance was solved by consensus with a third author (A.S.). Due to the rarity in literature of these types of complications, case reports and articles not written in English were also included in the review. Searches were limited to human studies and systematic reviews were excluded Table 1.

**Table 1:**
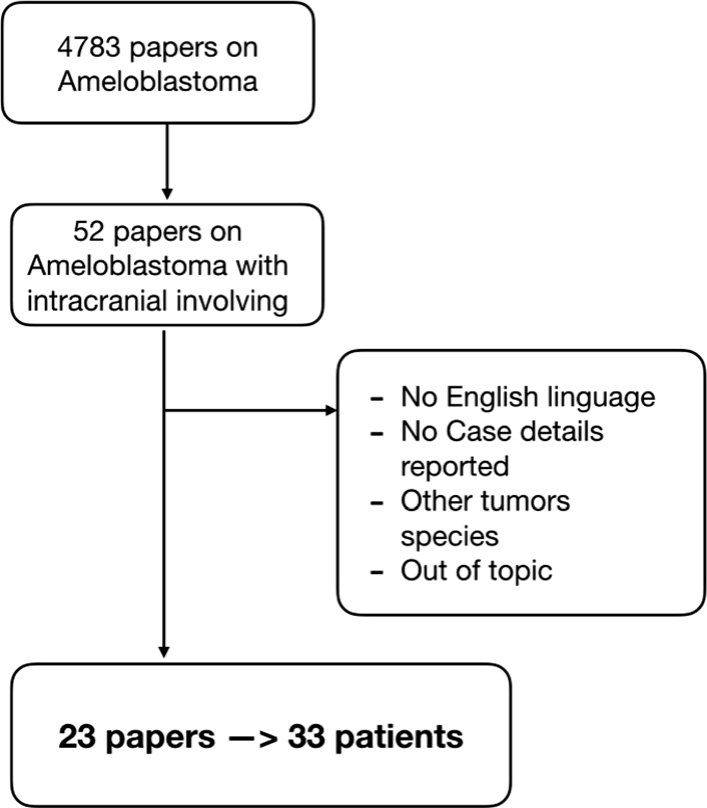
Flow chart of Study Selection. Systematic review was conducted in adherence to PRISMA guidelines

The main search returned a total of 4783 papers on AMBL as a topic. The details were collected: authors and year of publication, the number of patients, race, age, sex, the site of origin at first diagnosis of AMBL, if local recurrence was reported before the diagnosis of intracranial tumor involvement, the site of intracranial involvement, the time between the first diagnosis of AMBL (expressed in years) and the detection of the pathology at the intracranial level, if neurosurgery has been performed on the tumor, whether patients performed adjuvant RT, finally if there was the radiological finding of distant metastases, recurrence and the final prognosis of the patients (Table 2).

**Table 2:** Characteristics of included studies

No	Authors	No of patients	Race	Age	Sex	Site of origin	Histology	Local recurrence	Cranial Involvement	Latency (years)	Surgery cranial	Radiation Therapy	Other metastasis	Recurrence	Outcome
1	Harrer 1970 [29]	1	Caucasian	52	F	Maxillary	\	No	Anterior cranial fossa	23	No	No	Yes (Lung)	No	Dead 6 month
2	Kyriazis et. al. 1971 [30]	1	Caucasian	73	M	Maxillary	Follicular	Yes	Orbit	5	No	No	No	No	Dead 1 year
3	Shaw and Katsikas 1973 [31]	1	\	81	M	Maxillary	\	No	Orbit	0	No	50 gy	No	Yes	Dead 4 year
4	Daramola et. Al 1980 [32]	1	\	22	M	Maxillary	Follicular	Yes	Orbit	5	Yes	36 gy	Yes (lung)	No	Dead
5	Komisar et. al. 1984 [25]	1	\	63	M	Maxillary	Plexiform	Yes	Orbit, Anterior cranial fossa	2	No	No	No	No	Dead
6	Weiss et. al. 1985 [33]	1	\	72	M	Maxillary	Mixed	Yes	Orbit, Middle cranial fossa	5	Yes	No	No	Yes (1 year)	Dead
7	Oka et. Al. 1986 [34]	1	Asian	27	M	Mandibular	\	Yes	Middle cranial fossa	18	Yes	No	No	Yes (4 years)	Dead 1 year
8	Bredenkamp et. al. 1989 [4]	5	African	53	M	Maxillary	Mixed	No	Middle cranial fossa	0	No	64 gy	No	No	Alive
			Caucasian	59	F	Maxillary	Plexiform	No	Orbit	0	Yes	No	No	No	\
			Caucasian	43	M	Maxillary	Follicular	Yes	Middle cranial fossa	13	Yes	No	No	Yes	Alive
			Caucasian	15	M	Maxillary	Plexiform	Yes	Orbit	2	Yes	65 gy	No	Yes (3 years)	Dead 1 year
			Caucasian	36	F	Nasal	\	No	Anterior cranial fossa	2 months	Yes	No	No	No	Alive
9	Eliasson et. Al. 1989 [35]	1	\	40	F	Maxillary	\	Yes	Orbit, Middle cranial fossa	\	\	\	Yes (Lung)	Yes (8,5 year)	Alive
10	Scaccia et. al. 1991 [36]	2	Caucasian	53	M	Maxillary	\	Yes	Orbit, Middle cranial fossa	13	Yes	No	No	Yes (2 years)	Alive
			Caucasian	60	M	Maxillary	Follicular	No	Orbit	3	Yes	No	No	No	Alive
11	Phillips et. al. 1992 [10]	1	Caucasian	65	M	Mandibular	Mixed	Yes	Parieto-occipital	15	Yes	Yes	No	Yes (1 years)	\
12	Sato et. al. 1994 [37]	1	Asian	79	M	Maxillary	Follicular	Yes	Orbit, Anterior cranial fossa	9	Yes	No	No	No	Alive
13	Hayashi et. al. 1997 [38]	1	Asian	63	F	Mandibular	\	Yes	Orbit, Middle cranial fossa	27	Yes	29 Gy	No	No	Alive
14	Zarbo et. al. 2003 [39]	1	African	14	F	Maxillary	Spindle cell	Yes	Anterior cranial fossa	29	No	No	No	No	Dead 1 month
15	Goldenberg et. al. 2004 [40]	2	Caucasian	77	F	Mandibular	\	Yes	Not specified	10	Yes	\	No	\	\
			Caucasian	37	F	Maxillary	\	Yes	Anterior cranial fossa	3	No	Yes	No	\	Dead 1 month
16	Leibovitch et. al. 2006 [41]	2	Caucasian	73	M	Maxillary	Follicular	Yes	Middle cranial fossa	30	Yes	No	No	\	\
			Asian	60	F	Mandibular	\	Yes	Not Specified	7	Yes	Yes	No	\	\
17	Chen et. al. 2006 [27]	3	Asian	42	F	Mandibular	Mixed	Yes	Orbit, Anterior cranial fossa	15	Yes	No	No	Yes (6 years)	Alive
			Asian	57	F	Maxillary	Follicular	Yes	Fronto-temporal	13	Yes	No	No	Yes (4 years)	Dead
			Asian	34	F	Mandibular	Follicular	Yes	Fronto-temporal	17	Yes	No	No	Yes (3 years)	Alive
18	Yoshida et. Al. 2009 [42]	1	Asian	70	F	Maxillary	\	Yes	Fronto-temporal	\	\	\	\	\	\
19	Woodroffe et. al. 2013 [9]	1	Caucasian	60	M	Maxillary	\	Yes	Clivus	4	Yes	65 Gy	No	1 year	Alive
20	Rotellini et. al. 2016 [43]	1	Caucasian	29	M	Maxillary	Plexiform	Yes	Orbit, Middle cranial fossa	20	Yes	60 Gy	No	8 months	Alive
21	Quick-Weller et. al. 2016 [44]	1	African	57	M	Maxillary	\	Yes	Anterior cranial fossa	12	Yes	No	No	No	Alive
22	Bettoni et. Al. 2018 [45]	1	African	22	M	Mandibular	Papillary	Yes	Right parietal lobe	4	Yes	No	No	No	Alive
23	Li et. Al. 2019 [46]	1	Asian	28	F	Mandibular	\	Yes	Left frontal lobe	5	No	SRS 24,2 gy	Yes (lung)	\	\

## Results

A total of 32 cases were included in the qualitative analysis. The series consisted of 18 males and 15 females, and the mean age was 50 years. It is no clear a predominance of African rather than European or Asian individuals in the occurrence of this disease course (4 Africans—14.3%, 9 Asiatic—32.1% and 15 Caucasic—53.6%). 23 out of 33 patients (69.7%) had the maxillary bone as the first site of origin of the tumor against 9/33 (27.3%) patients who showed the first site the mandibular bone and one patient who had shown the nasal bones as the origin of the tumor (3%). The histological report was reported in series on 19 patients, where 9 patients were diagnosed with follicular AMBL (47.4%), 4 AMBL mixed and AMBL Plexiform (21.1% respectively), 1 papillary form and 1 spindle-cell form (5.26% respectively). Before the radiological diagnosis of intracranial involvement of the tumor, patients reported the diagnosis and treatment of a local recurrence of primary AMBL in 27/33 cases (81.8%). The most frequent site of involvement was the orbit in 15/31 patients (48.4%), followed by the anterior cranial fossa floor 9/31 (29%) and mean 9/31 (29%). The cases in which there was no direct spreading of the lesion but a multifocal area distant from the site of origin was diagnosed is 7/31 cases (22.6%). The average estimated time for the detection of an intracranial lesion from AMBL is about 10 years with cases in which the finding was contextual (synchronous metastasis 3/33 cases—9%), short term (1 patient with 2 months finding) and a maximum of 30 years. Information on surgical treatment is available on 31/33 cases. It was possible to perform a radical surgical removal in 23/31 patients (74.2%), while for 8/31 cases (34.8%) it was only possible to biopsy the lesion to obtain a certain diagnosis. The reasons reported were the difficulty of planning the intervention (diagnostic impossibility), the patient's general clinical status and the patient's will. In 13/31 (41.2%) patients, disease recurrence was found at the intervention site, with a reported survival rate of 15/31 cases (48.4%) It was not possible to retrieve a statistically significant analysis of the studies reported.

## Clinical Case

A 56-year-old woman presented with a history of recurrent left maxilla AMBL with multiple recurrences of a maxillary follicular AMBL that developed direct intracranial extension with dural involvement of the anterior and medial cranial fossa. The endonasal endoscopic assisted and open combined approach was performed with a successful complete tumor resection. Patient was treated in our institution in February 2019.

**Fig. 1:**
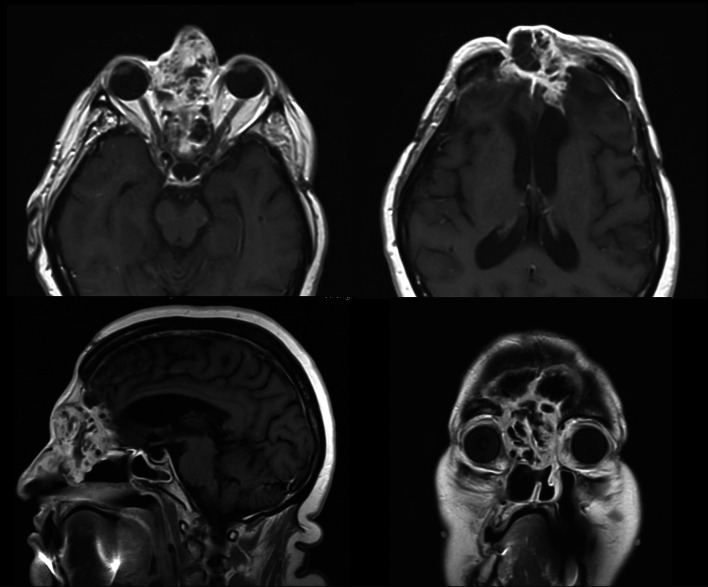
MRI images in axial, sagittal and coronal views showing intracranial infiltration of the AMBL

Previous medical history: the disease's onset was five years ago with a history of facial pain, mild gaze palsy, decreased left facial sensation, and speech and swallowing changes. She was evaluated and treated at first by an odontologist who diagnosed a presence of AMBL invading the maxillary sinus. The initial tumor resection was performed in 2015 and consisted of a left high maxillectomy. The patient was closely monitored. The initial tumor recurrence has been noted on follow-up CT scans in the left posterior ethmoid after 6 months. Given the extensive local invasion of the lesion and new intracranial extension, a combined approach was planned to maximize cytoreduction. An image-guided, endoscopic transnasal approach was utilized with posterior septectomy and right sphenoidotomy. The histological analysis confirmed a recurrence of AMBL with follicular components a complete resection was performed with surgical soft tissue margins superior to 1 cm. A second recurrence located on the ethmoid and frontal sinus was found two years later. The extension was then further assessed by magnetic resonance imaging (MRI) and revealed an osteolytic soft tissue lesion with an extradural component resulting in a mass effect on the left and right cerebral frontal lobe (Fig. 1), without any synchronous other lesions (lymphatic or pulmonary) objectified on the full-body CT scanner.

**Fig. 2:**
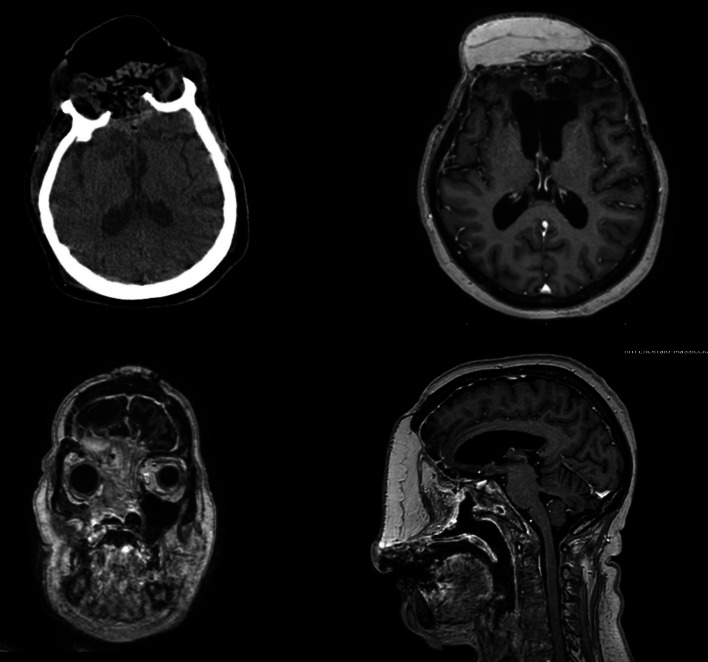
Postoperative MR images in axial, coronal and sagittal views showing the free flap, skin, subcutaneous and muscular layers in the absence of local recurrence of the AMLB

**Fig. 3:**
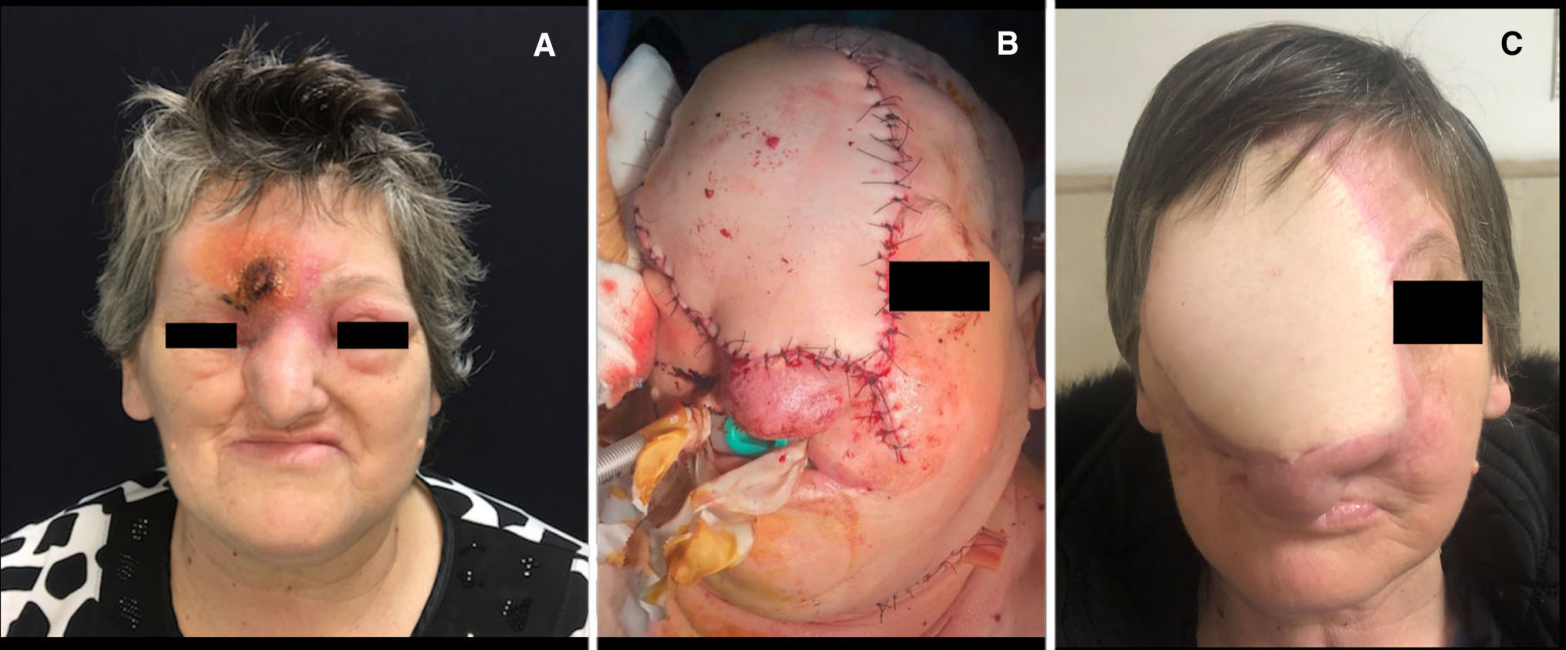
Image A: preoperative front view of the patient showing skin infiltration of the AMBL. Image B: immediate postoperative view, presence of the endotracheal tube. Image C: stable postoperative at 12 months of follow-up

In 2019, the patient was finally admitted to our Institution, Human Neurosciences Department Neurosurgery Division, Sapienza University of Rome and treated through a combined surgical approach with a multidisciplinary team, neurosurgeons and maxillo-facial surgeons. Taking into account the evolutionary nature of the lesion and the potential brain damage, a radical approach was performed with complete surgical resection of this ameloblastic lesion, followed by skull reconstruction using cement covered with a latissimus dorsi myocutaneous free flap. A bicoronary skin incision was used and an isolated pericranial flap and a dural synthetic patch were prepared. The operation consisted of a classical bifrontal trepanation, removal of the solid tumor mass frontobasal to the level of the eroded bone of the cranial base, and an enlargement of the opened dura mater to remove the neurocranial tumor parts. The tumor appeared as a white mass and seemed not to be very vascularized. Removal was enlarged until both frontal lobes were visible. The tumor was carefully resected by the preparation of the border zone between the cortex and the lesion. A dura substitute was sewn in to cover the brain, and a Spongostan (Ethicon, Norderstedt, Germany) and Duragen (Integra LifeSciences Corp., Plainsboro, New Jersey, United States) layer was applied before the galea patch were used to cover the intracranial area. The second part of the procedure consisted of a combined bifrontal and midfacial approach, exenteration of the right orbit, and extirpation of the perisinusoidal- and retropharyngeal tumor masses. The tumor mass was then resected en-bloc. A latissimus dorsi myo-cutaneous free flap was raised and used to fill the residual dead space. A subcutaneous tunnel in left parotid region was realized to allow the pedicle to reach the facial vessels and perform the anastomosis. Every step was accompanied by histological analysis of every resected margin, frozen margins were negative. The postoperative course of the intracranial surgery was uneventful. After 3 days in the intensive care unit (ICU), the patient was discharged to the normal ward. The patient developed no neurologic deficits postoperatively. A Contrast-enhanced brain MRI scan, performed the following day, did not demonstrate any residual enhancement. Nine days after, the patient was discharged. During the hospitalization no rhinoliquorrhea or otholiquorrhea were observed also. Six months after the operation, follow-up imaging remained stable with no dural enhancement. Postoperative follow-up MRI at 12 months showed no enhancing lesions to suggest tumor recurrence (Fig. 2); At her most recent clinic visit, 18 months postoperatively, the patient had no change in symptoms to suggest disease recurrence. At the time of writing, the patient remains clinically stable, has a regular follow-up, and waiting for plastic mid facial reconstruction surgery (Fig. 3).

## Discussion

AMBL is a benign odontogenic tumor but the high local recurrence rate and intracranial involvement make the management controversial. In a study of 315 AMBL patients published by Olaitan et al. [7], only three patients (< 1%) showed an intracranial manifestation, but in the largest study reviewing patients with malignant AMBL of all types between 1966 and 1995 found that approximately 10% had brain involvement, by direct extension [8]. The last complete review of the literature dates back to 1989 with Bredenkamp et. [4]. Since then, only Woodroffe et. [9] in 2014 last published a short collection which was incomplete in the number of cases and the variables considered. This topic's rarity is not so much in the pathology but rather in the intracranial involvement of the lesion and subsequent surgical management [10, 11]. Only 32 cases are reported in the literature in which the intraoperative and postoperative management is varied and often not described, resulting in an almost complete lack of information and indications for treatment. There are multiple approaches for the management of AMBL. Radical surgery tends to yield the best outcomes, and it is recommended to have adequate surgical margins when possible. Debate exists on the degree of margins recommended, but it ranges from 1.0 to 3.0 cm [12–15]. So, radical surgical treatment is considered customarily the treatment of choice for a biologically aggressive subtype of primary and recurrent AMBL. It involves en-bloc tumor resection with a wide bone margin followed by immediate or delayed bone reconstruction of the surgical defect with tissue grafts and prosthetic rehabilitation [16]. Higher incidences are found in Africa, China, and India compared to the Western World, but if we consider the patterns of the tumor's aggression, it seems that there are no geographic epidemiological differences. Although some clinical studies do not demonstrate a predilection for sex, race, or age [17], it seems to be a difference in the median age of the first diagnosis; it is 24 years in developing countries and 38 years in industrialized countries [12]. Interestingly, there is little evidence that histologic grade influences local extension and intracranial spread [18]. Four different types of AMBL are described in the WHO classification for odontogenic tumors [12]: The solid/multicystic type is the most common and accounts for 90% of all AMBLs. Within this group, the plexiform and the follicular histological patterns are found most frequently. The follicular type can show different cytological differentiation, such as granular, basal cell, and spindle cell types [19–21]. Histopathologic examination of the present collection showed a predominance of follicular type tumor, [22], where epithelial islands simulate enamel organs and often mimic the appearance of regular ameloblasts [12]. Also, follicular AMBLs seem to have a higher recurrence rate than the others variety [12, 22, 23] and have a propensity to invade the cranial vault, and this most likely relates to anatomical proximity rather than some inherent difference in tumor biology [2]. Risk factors for AMBL with intracranial involvement include the volume of the primary lesion, site, delayed diagnosis of the initial tumor, multiple local recurrences, inadequate surgical resection, a previous history of radiation/chemotherapy treatment, and plexiform histology [24]. Duration of disease and an increasing number of recurrences appear to be risk factors for direct intracranial involvement [9]. The presence or absence of other metastases is not indicative of an increased risk of developing brain disease. The maxillary bone's spongy architecture facilitates the tumor's spread [17], and direct extension may lead to brain involvement. Maxillary AMBLs are inherently more destructive than those within the mandible. They are also more difficult to treat due to the maxilla's thin bone, which allows relatively unrestricted growth [4]. Sometimes a gross total surgical resection for these patients is not indicated due to the extent of disease [4]; but they treated conservatively with excision of only tumor-involved hard and soft tissue have both a higher rate of recurrence [25, 26] and earlier recurrence [12] than those treated with radical surgery. It should be noted that almost all patients before having the intracranial compartment's involvement show signs of local recurrence amenable to treatment, and many of them at the time of the first diagnosis of recurrence already have cerebral involvement. This data justify the higher average age of onset (50 years) and the wide variability of the cerebral pathological manifestation time. A local recurrence could be possible regarding the loco-regional development despite histologically negative margins by ameloblastic cells found in muscle tissue and resulting from dissemination during surgery [27, 28]. Histologically, tumors invade intertrabecular spaces and may not be visible grossly or radiographically, underscoring the need for radical resection; for this reason, we sustain the importance of histological extemporary analysis during asportation. It is widely considered that complete tumor excision yields the best opportunity for disease cure, and it may be that recurrence reflects failure or inadequacy of the primary surgical resection [12, 15, 17]. Our review appears to be the most complete ever published; the collection of case histories may not be considered complete due to many cases not described. Some of them are reported in cases from a few decades ago, in an era in which modern MRI diagnostics and modern technologies introduced in radiotherapy were not yet present. However, we believe it is appropriate to diagnose recurrent ameloblastoma never to consider the radiological follow-up concluded and that an MRI study involving the brain and a total-body CT is always indicated. There is a wide spectrum of treatment outcomes found in the literature, and given the rarity of AMBL, evidence-based findings are lacking. [9] After a tumor progresses past the point where complete resection is possible, patients will typically undergo repeated debulking with adjuvant radiation. Once disease surrounds vital structures, treatment is generally unsuccessful, and the intent of surgery becomes palliative rather than curative [4, 26]. While not known to be curative, chemotherapy and radiation are often used as palliative treatment to manage residual disease [12], as in the present patient. These therapies should be reserved as primary treatment modalities only when surgery is not possible. The interrelationship between the clinical and histological properties of the AMBL determines its aggressiveness, which dictates the treatment approach and recurrence. However, aggressive treatment is always related to potential facial deformity and the psychological effect and on the reduction in the Quality-of-Life of the patient after surgery. In conclusion, the most radical treatment possible and a close neuroradiological follow-up, almost as if we were facing a malignant pathology, must be applied when the AMBL has these characteristics: recurrence, even if local, involvement of the upper jaw close to the cranial fossa and the follicular type. In this case, it is also strictly necessary to monitor intracranial spreading and distant metastases during follow-up.
